# Changes in Cervical Human Papillomavirus (HPV) Prevalence at a Youth Clinic in Stockholm, Sweden, a Decade After the Introduction of the HPV Vaccine

**DOI:** 10.3389/fcimb.2019.00059

**Published:** 2019-03-20

**Authors:** Andreas Ährlund-Richter, Liqin Cheng, Yue O. O. Hu, Mikaela Svensson, Alexandra A. L. Pennhag, Ramona G. Ursu, Linnea Haeggblom, Nathalie Grün, Torbjörn Ramqvist, Lars Engstrand, Tina Dalianis, Juan Du

**Affiliations:** ^1^Department of Oncology and Pathology, Karolinska Institutet, Stockholm, Sweden; ^2^Department of Microbiology, Tumor and Cell Biology, Centre for Translational Microbiome Research, Karolinska Institutet, Stockholm, Sweden; ^3^Science for Life Laboratory, Department of Microbiology, Tumor and Cell Biology, Stockholm, Sweden; ^4^Department of Microbiology (Bacteriology, Virology) and Parasitology, Grigore T. Popa University of Medicine and Pharmacy, Iaşi, Romania

**Keywords:** HPV—human papillomavirus, cervical HPV-prevalence, HPV-vaccines, youth clinic, women

## Abstract

**Aim:** This study aimed to follow the impact of human papillomavirus (HPV) catch-up and vaccination on the very high cervical HPV-prevalence in women at a youth clinic in central Stockholm during the period 2008–2018.

**Background:** 2008–2010, cervical HPV-prevalence (69.5%) and HPV16 prevalence (34.7%) were high in non-vaccinated women at a youth clinic in Stockholm. 2013–2015, after the introduction of the quadrivalent-Gardasil® HPV-vaccine, HPV16 and HPV6 prevalence had decreased. Here, cervical HPV-prevalence was investigated 10 years after primary sampling.

**Material and Methods:** 2017–2018, 178 cervical swabs, from women aged 15–23 years old, were tested for 27 HPV types by a bead-based multiplex method. HPV-prevalence data were then related to vaccination status and age and compared to HPV-prevalence in 615 samples from 2008 to 2010 and 338 samples from 2013 to 2015 from the same clinic, and to HPV types in 143 cervical cancer cases during 2003–2008 in Stockholm.

**Results:** The proportion of vaccinated women increased from 10.7% (2008–2010) to 82.1% (2017–2018). The prevalence of all 27 HPVs, all high-risk HPVs (HR-HPVs) and the combined presence of the quadrivalent-Gardasil® types HPV16, 18, 6, and 11, was lower in vaccinated compared to unvaccinated women (67.4 vs. 93.3%, *p* = 0.0031, 60.1 vs. 86.7%, *p* = 0.0057 and 5.8 vs. 26.7%, *p* = 0.002, respectively). Furthermore, HPV16 prevalence in non-vaccinated women 2017–2018 was lower than that in 2008–2010 (16.7 and 34.7%, respectively, *p* = 0.0471) and similar trends were observed for HPV18 and 11. In both vaccinated and non-vaccinated women, the most common non-quadrivalent-Gardasil® vaccine HR-HPV types were HPV39, 51, 52, 56, and 59. Together they accounted for around 9.8% of cervical cancer cases in Stockholm during 2003–2008, and their prevalence tended to have increased during 2017–2018 compared to 2008–2010.

**Conclusion:** Quadrivalent-Gardasil® vaccination has decreased HPV-vaccine type prevalence significantly. However, non-vaccine HR-HPV types remain high in potentially high-risk women at a youth clinic in Stockholm.

## Introduction

Human papillomavirus (HPV) infections represent the most common sexually transmitted diseases, affecting millions of people worldwide. About 90% of HPV infections are cleared within 2 years without any need for medical intervention (Ho et al., 1998). However, persistent infections with high-risk HPV (HR-HPV) types can progress to cancer (Ho et al., 1998; Tommasino, 2014). Low-risk HPV (LR-HPV) types do not usually cause cancers but are associated with the occurrence of genital warts and respiratory papillomatosis (Ho et al., 1998; Tommasino, 2014). There is also evidence that links HPV with other types of cancer, e.g., vaginal, vulvar, penile, anal, as well as oropharyngeal cancer, especially tonsillar and base of tongue cancer (Forte et al., 2012; Dunne and Park, 2013; Tommasino, 2014; Näsman et al., 2017).

The US Food and Drug Administration (FDA) has approved three HPV-vaccines: quadrivalent-Gardasil®, Cervarix®, and Gardasil®9, consisting of type-specific HPV L1 virus-like particles (VLPs) that induce type-restricted protection (Schiller and Lowy, 2018). All three vaccines prevent HPV16 and HPV18 infection, which account for about 70% of cervical cancer and precancerous cervical lesions (Koutsky et al., 2002; Schiller and Lowy, 2018). Quadrivalent-Gardasil® also protects against HPV6 and HPV11, which cause 90% of genital warts (Schiller and Lowy, 2018). Gardasil®9 targets an additional five cancer-causing HPV types (HPV31, 33, 45, 52, and 58) (Schiller and Lowy, 2018). These vaccines may also have some cross-protection against other less common HR-HPV types (Kavanagh et al., 2017). Since the first licensure of HPV-vaccination in 2006, many countries have implemented publicly funded HPV-vaccination programs, with a variety of observational studies documenting vaccine efficacy and impact (Gallagher et al., 2018). HPV-vaccination is documented to be safe, immunogenic, and associated with decreased HPV infection rates and lowered risk of HPV related diseases (Drolet et al., 2015; Sankaranarayanan et al., 2016; Niccolai et al., 2017). Seven years (2007–2014) follow-up of the female three-dose HPV-vaccine series in nine high-income countries demonstrated a 68% decrease in the incidence of HPV16 and HPV18 (Drolet et al., 2015).

In Sweden, HPV-vaccination was gradually introduced and subsidized between 2006 and 2011 for 13–17 years old girls, but initially with a fairly low coverage (Vänskä et al., 2018). In 2012, the quadrivalent-Gardasil® vaccine was introduced into the school-based vaccination program for 10–12 years old girls. Meanwhile a catch-up vaccination included young women up to 26 years of age in Stockholm, Sweden, which increased the total vaccine coverage (Vänskä et al., 2018).

In 2008–2010, when catch-up vaccination was still low (10.7%), we reported a cervical HPV-prevalence of 69.5% (with 34.7% infected by HPV16, and 10.1% infected by HPV18, respectively) in HPV non-vaccinated individuals at a youth clinic in Stockholm, Sweden (Ramqvist et al., 2011). In a follow-up study performed 2013–2015 at the same youth clinic, we showed that HPV-vaccination and catch-up vaccination coverage had increased to 71.0% and correlated with a significant decrease in cervical prevalence of HPV16 and HPV6 both covered by the quadrivalent-Gardasil® vaccine (Grün et al., 2016). However, very few studies focus on HPV types not targeted by the present HPV-vaccines. Given the very high pre-vaccine prevalence of HR-HPV infection in young women at the Stockholm youth clinic, it was of special interest to further assess the current coverage of the vaccination program and to evaluate the impact of HPV-vaccination on cervical HPV-prevalence one decade after our first survey (Ramqvist et al., 2011).

We therefore followed up young women visiting the same youth clinic in 2017–2018 and analyzed cervical prevalence of 27 different HPV types. Obtained data were then compared to data acquired from samples collected 2008–2010 and 2013–2015 during the introduction of the HPV-vaccination program the past decade (Ramqvist et al., 2011; Grün et al., 2016). In addition, we investigated the influence of school-based HPV-vaccination program and catch-up vaccination on the prevalence of the four vaccine types as well as non-vaccine HR-HPVs. Finally, HPV-prevalence was compared according to vaccination status and between different age groups and compared to HPV-prevalence found 2003–2008 in cervical cancer in the Stockholm region (Du et al., 2011). This study therefore provides an overall map of the influence of school-based HPV-vaccination program and catch-up vaccination program during the period of 2008–2018 among the potentially high-risk women, in Stockholm, Sweden.

## Materials and Methods

### Study Population

In total 178 self-collected cervical swabs and information of vaccination status and age were obtained from women aged 15–23 years between January 2017 and June 2018 at a youth clinic in central Stockholm, Sweden. Participation of this study was anonymous. Participates were informed and written consents were obtained before sample collection. Most young women were either HPV-vaccinated or catch-up vaccinated through the Swedish national vaccination program with the quadrivalent-Gardasil® vaccine against HPV16, 18, 6, and 11. The data acquired were then compared to data obtained at the same youth clinic in 2008–2010 (615 samples, 10.7% vaccination coverage) and 2013–2015 (338 samples, 71.0% vaccination coverage) and to HPV types occurring 2003–2008 in 143 cervical cancer cases (111 squamous carcinomas and 32 adenocarcinomas), in Stockholm (Du et al., 2011; Ramqvist et al., 2011; Grün et al., 2016). This study was performed according to permissions 2008/813-31/2, 2008/870-31/4, and 2017/725-31 approved by Stockholm Regional Ethics Committee.

### Sample Collection and DNA Extraction

Sample collection was performed as described before (Ramqvist et al., 2011). However, both the sampling buffer and DNA extraction method were modified, the latter now performed as reported by Hugerth et al. (2018). Briefly, cervical swabs preserved in DNA/RNA Shield (Zymo Research Corp, Irvine, CA) where beads were beaten with ZR Bashing Bead Lysis Tubes (0.1 and 0.5 mm from Zymo Research Corp, Irvine, CA) at 1,600 rpm with the 96 FastPrep machine (MP Biomedicals, Santa Ana, CA, USA) for 1 min. The sample buffer was then spun at 4,400 rpm for 4 min to separate out the beads. Thereafter, the supernatant was incubated with lysozyme buffer (20 mM Tris-Cl, 2 mM sodium-EDTA, 100 g/ml lysozyme; Sigma, St. Louis, MO, USA) at 37°C for 3 h while shaking at 1,000 rpm. The DNA extraction was then conducted with the ZR-96 Genomic DNA MagPrep kit (Zymo Research Corp, Irvine, CA) according to the manufacturer's instruction. Finally, the extracted DNA was eluted from the magnetic beads with 70 μl Elution Buffer (10 mM Tris-Cl, pH 8.5; Qiagen, Venlo, Netherlands) and the purified DNA was stored at −20°C before HPV genotyping. For each extraction, DNA-free water samples were included as negative controls for potential contamination.

### HPV Genotyping

In total, 27 subtypes of HPV were assayed for by a multiplex bead-based assay, as reported before (Schmitt et al., 2006; Ramqvist et al., 2011; Nordfors et al., 2014; Dalianis et al., 2015). These included 15 HR-types (HPV16, 18, 31, 33, 35, 39, 45, 51, 52, 56, 58, 59, 68, 73, 82), 5 potentially HR-types (26, 30, 53, 66, 67 and 69), and 6 LR-types (HPV6, 11, 42, 43, 44, and 70) (Schmitt et al., 2006; Bihl et al., 2017). Notably, typing of HPV30, 67, and 69 was not included in the corresponding assays of the initial sample collection 2008–2010 from the youth clinic or the 2003–2008 cervical cancer sample collection (Du et al., 2011; Ramqvist et al., 2011). Briefly, for samples tested 2017–2018, PCR amplification with broad-spectrum BGP5^+^/6^+^ primers targeting the L1 region was carried out on 5 μl of DNA in a 25 μl reaction mix containing biotinylated primers from Qiagen Multiplex PCR Master Mix (Qiagen, Hilden, Germany). For HPV16 and HPV33 primers and probes targeting the E6-1F region were also included as described previously (Nordfors et al., 2014; Dalianis et al., 2015). The samples were analyzed by a MAGPIX instrument (Luminex Inc., TX, USA) in 96 well plates following manufacturer's instructions. Samples positive for L1 (and/or E6 for HPV16 and 33) were regarded as HPV-positive. For each run, a positive control and negative extraction control were added. Values with only water, before the PCR run were considered as background. The cut-off was set as: raw median fluorescent index (MFI) – 15 – 1.5 × background, except for the following cases: Firstly, a higher cut for HPV33-L1, HPV16-L1, HPV59, 69, and HPV82 (MFI – 25 – 1.5 × background) were used due to a higher background variation. Secondly, for HPV82 a cut-off of: MFI-125 – 1.5 × background, in cases with an HPV51 value >100, due to a potential cross-reaction.

### Statistical Analysis

Any HPV infection/overall HPV infection, defined as all 27 HPV types, HR-HPV infection defined as the 15 HR-HPV types, infection with quadrivalent-Gardasil® HPV types (HPV16, 18, 6, and 11) together or alone, were compared between vaccinated and unvaccinated groups using Chi-square analysis. Differences of HPV types collected from this study were also compared with former studies using Chi-square analysis.

## Results

### HPV Cervical Prevalence 2017–2018 Is High in Women Irrespective of Vaccination Status

**Figure 1:**
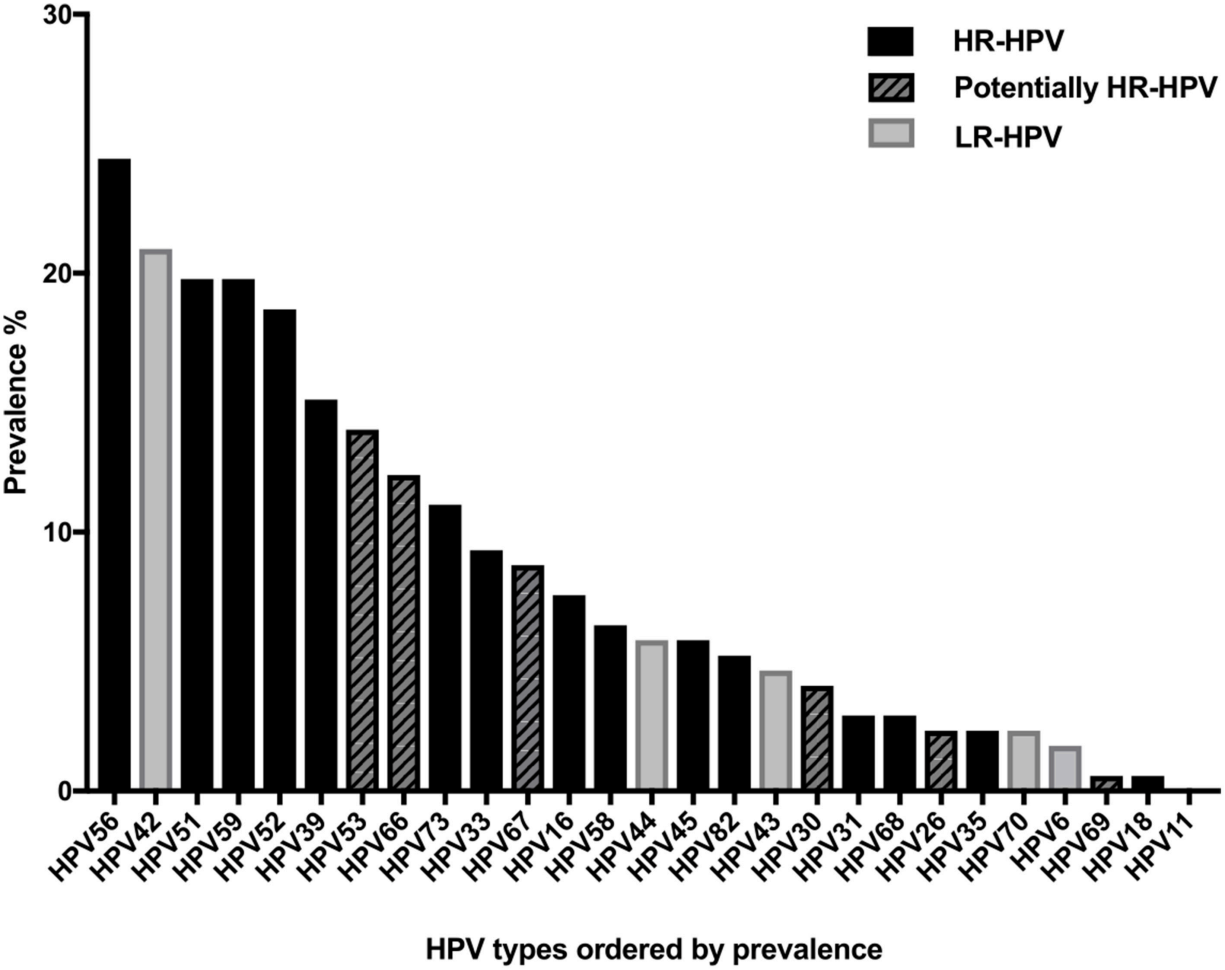
Cervical human papillomavirus (HPV) prevalence of all tested HPV types irrespective of vaccination status.

In total, 172/178 samples had sufficient material for HPV analysis. Total cervical HPV-prevalence, irrespective of HPV-vaccination status, was 72.1% (124/172) and HR-HPV prevalence was 65.1% (112/172) (data not shown). Figure 1 displays all HPV types in HPV-prevalence order. The five most common HR-HPV types: HPV56 (24.4%), HPV51, HPV59 (both 19.8%), HPV52 (18.6%), and HPV39 (15.1%) were all none quadrivalent-Gardasil® vaccine types. The prevalence of the quadrivalent-Gardasil® vaccine types HPV16, HPV18, HPV6, and HPV11, were 7.6, 0.6, 1.7, and 0%, respectively. Most common LR-HPV and potentially HR-HPV types were HPV42, 53, 66, 67, and 44 (20.9, 14.0, 12.2, 8.7, and 5.8%, respectively).

### HPV Cervical Prevalence 2017–2018 Is Generally Lower in Vaccinated Women

**Figure 2:**
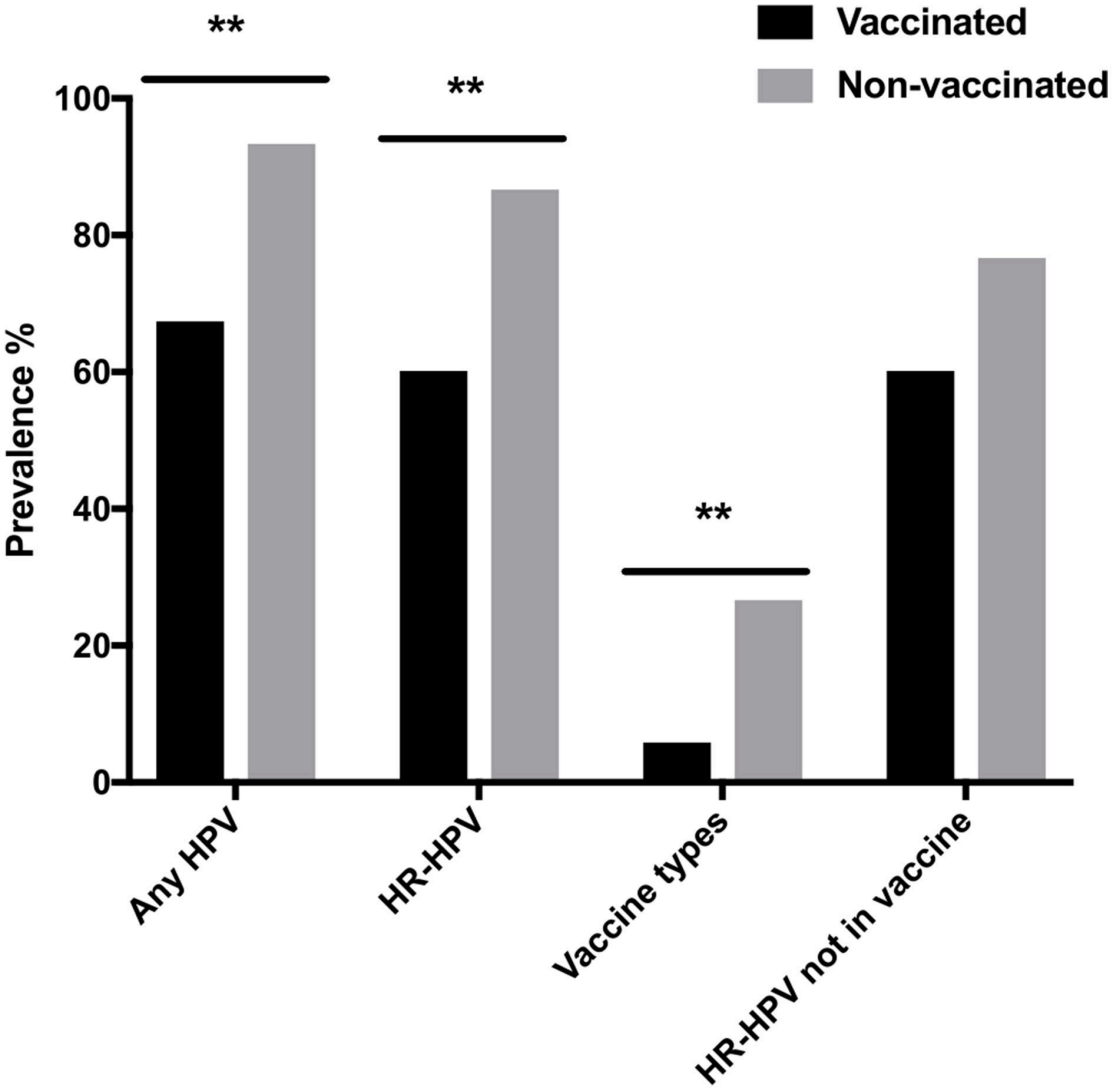
Cervical human papillomavirus (HPV) prevalence in vaccinated and non-vaccinated women with regard to: all 27 assayed HPV types (Any HPV); high-risk HPVs (HR-HPV); quadrivalent-Gardasil (R) vaccine types HPV16, 18, 6, and 11 (Vaccine types); and HR-HPVs not in the vaccine (HR-HPV not in vaccine). ^**^p < 0.01.

Vaccination status was available for 168/172 women above. Of these 138/168 (82.1%), had received at least one HPV-vaccination. Figure 2 shows HPV-prevalence in relation to vaccination status. Any HPV-prevalence (all 27 HPV types) and HR-HPV prevalence were both lower in vaccinated than in non-vaccinated women (67.4 vs. 93.3%, *p* = 0.0031 and 60.1 vs. 86.7%, *p* = 0.0057, respectively). The four specific HPV-vaccine types combined were also less common in vaccinated than in unvaccinated women (5.8 vs. 26.7%, *p* = 0.002), while for the 13 non-vaccine HR-HPV types the same trend was not statistically significant 60.1 vs. 76.7%, *p* = 0.099).

### HPV Type Specific Cervical Prevalence 2017–2018 According to Vaccination Status Show Significant Decreases of HPV6, 33, 45, and 44 in HPV-Vaccinated Women

**Figure 3:**
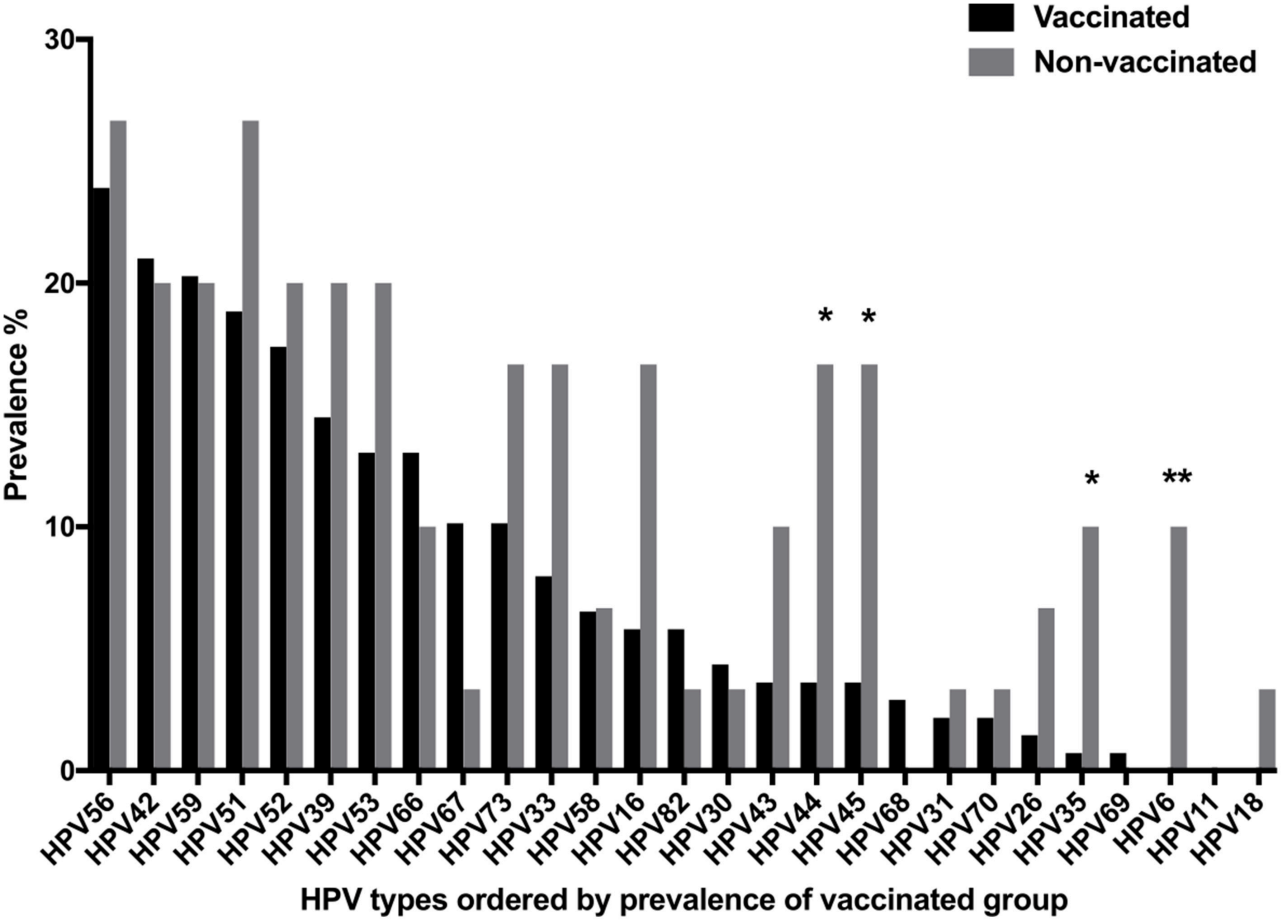
Cervical human papillomavirus (HPV) prevalence of all tested HPV types according to vaccination status (Vaccinated or non-vaccinated). ^*^p < 0.05, ^**^p < 0.01.

HPV type specific cervical prevalence according to vaccine status is presented in Figure 3. HPV16 and HPV18 prevalence, included in all HPV-vaccines, tended to be lower in HPV-vaccinated than in non-vaccinated women (5.8 vs. 16.7%, *p* = 0.058 and 0 vs. 3.3%, *p* = 0.179 respectively), but did not reach statistical significance (Figure 3). Quadrivalent-Gardasil® type HPV6 was significantly lower in vaccinated compared to non-vaccinated women (0 vs. 10.0%, *p* = 0.0052), while HPV11 was not detected at all (neither in vaccinated or non-vaccinated women) in this study (Figure 3).

Notably, the prevalence of both HR-HPV35 and 45 also decreased significantly (*p* = 0.0184 and *p* = 0.0172, respectively) in vaccinated as compared to non-vaccinated women, while this was not the case for the remaining HR-HPV types (Figure 3). The five most common non-vaccine HR-HPV types were HPV39, 51, 52, 56, and 59 irrespective of vaccination status (Figure 3). In vaccinated women, they had the following prevalence: HPV56 (23.9%), HPV59 (20.3%), HPV51 (18.8%), HPV52 (17.4%), and HPV39 (14.5%), and in non-vaccinated women corresponding figures were: HPV51 and 56 (both 26.7%) and HPV39, 52, and 59 (all 20.0%), with no significant differences between the two groups (Figure 3). There were no significant differences in four of the five most common non-HR-HPV types, HPV42, 53, 66, and 67, between vaccinated and non-vaccinated women. However, LR-HPV 44 was significantly less common in the vaccinated group, *p* = 0.0172 (Figure 3).

### Cervical HPV Prevalence According to Vaccination Status and Age

**Figure 4:**
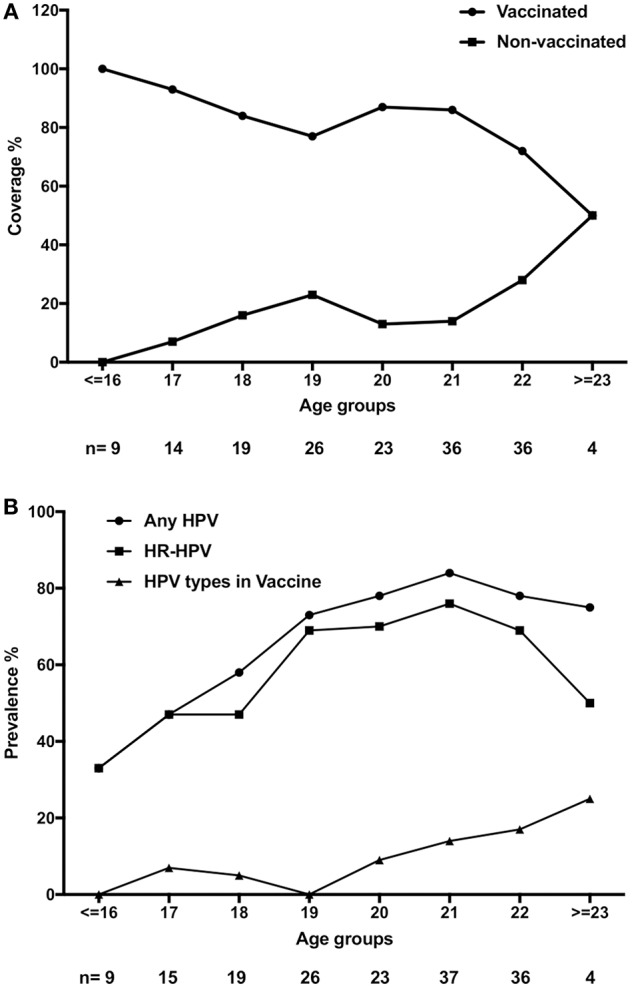
Vaccination status and human papillomavirus (HPV) status according to age. **(A)** HPV-vaccination status (Vaccinated and Non-vaccinated, respectively) according to age. **(B)** Presence of any of the 27 assayed HPV types (Any HPV), high-risk HPV (HR-HPV) and the combined prevalence of vaccine HPV types 16, 18, 6, and 11 (HPV types in vaccine) according to age.

Vaccine status and age were obtained from 167/172 women with cervical samples analyzed for HPV DNA. HPV-vaccination coverage was 100% in women ≤16 years and ~80% coverage in those ≤19 years (Figure 4A). There were 169 samples from women with information on HPV-prevalence and age (Figure 4B). Data from women ≤16 years, as well as ≥23 years were grouped together due to small sample sizes. Irrespective of vaccination status, HPV-prevalence increased from ≤16 years of age (33.3%), peaked at 21 years of age (83.8%), and then slowly decreased (77.8%) and HR-HPV types accounted for most cases, especially in younger women (Figure 4B). The prevalence of the HPV types covered by the vaccine was relatively low with <10% in those ≤20 years of age but tended to be somewhat higher (13.5–25.0%) in those >20 years of age (Figure 4B).

### HPV Types Per Cervical Sample 2017–2018

Most women had an infection with at least one HPV type, and mainly a HR-HPV type. The average number of any of the assayed 27 HPV types (any HPV) or 15 HR-HPV types per sample tended to be lower in vaccinated than in unvaccinated women (2.1 vs. 3.1 and 1.4 vs. 2.1, respectively). However, when only HPV infected women are considered, the average number of any HPV or HR-HPV type per sample was similar in vaccinated and non-vaccinated women (3.2 vs. 3.3 and 2.1 vs. 2.2, respectively). The maximum HPV types per sample was 11, (9/11 HR-HPVs), and was detected in a sample of a non-vaccinated woman.

### Cervical HPV-Prevalence 2017–2018 Compared to Previous Data From 2013 to 2015 and 2008 to 2010

HPV-vaccination coverage increased from 10.7% (2008–2010) to 71.0% (2013–2015) and 82.1% (2017–2018). Initially most women were HPV catch-up vaccinated, while in 2017–2018, most women aged 16–18 years were vaccinated, likely through the school-based vaccination program. HPV-prevalence was generally high irrespective of time period. More specifically, when comparing vaccinated with non-vaccinated women, it was 53.9 (earlier unpublished data) vs. 69.5% in 2008–2010, 64.6 vs. 74.5% in 2013–2015, and most recently in 2017–2018, it was 67.4 vs. 93.3% (Ramqvist et al., 2011; Grün et al., 2016). HR-HPVs infections were also common, in non-vaccinated women (61.6%) 2008–2010, and in vaccinated vs. non-vaccinated women, 50.8 vs. 62.2%, respectively in 2013–2015, and 60.1 vs. 86.7%, respectively in 2017–2018 (Ramqvist et al., 2011; Grün et al., 2016).

**Figure 5:**
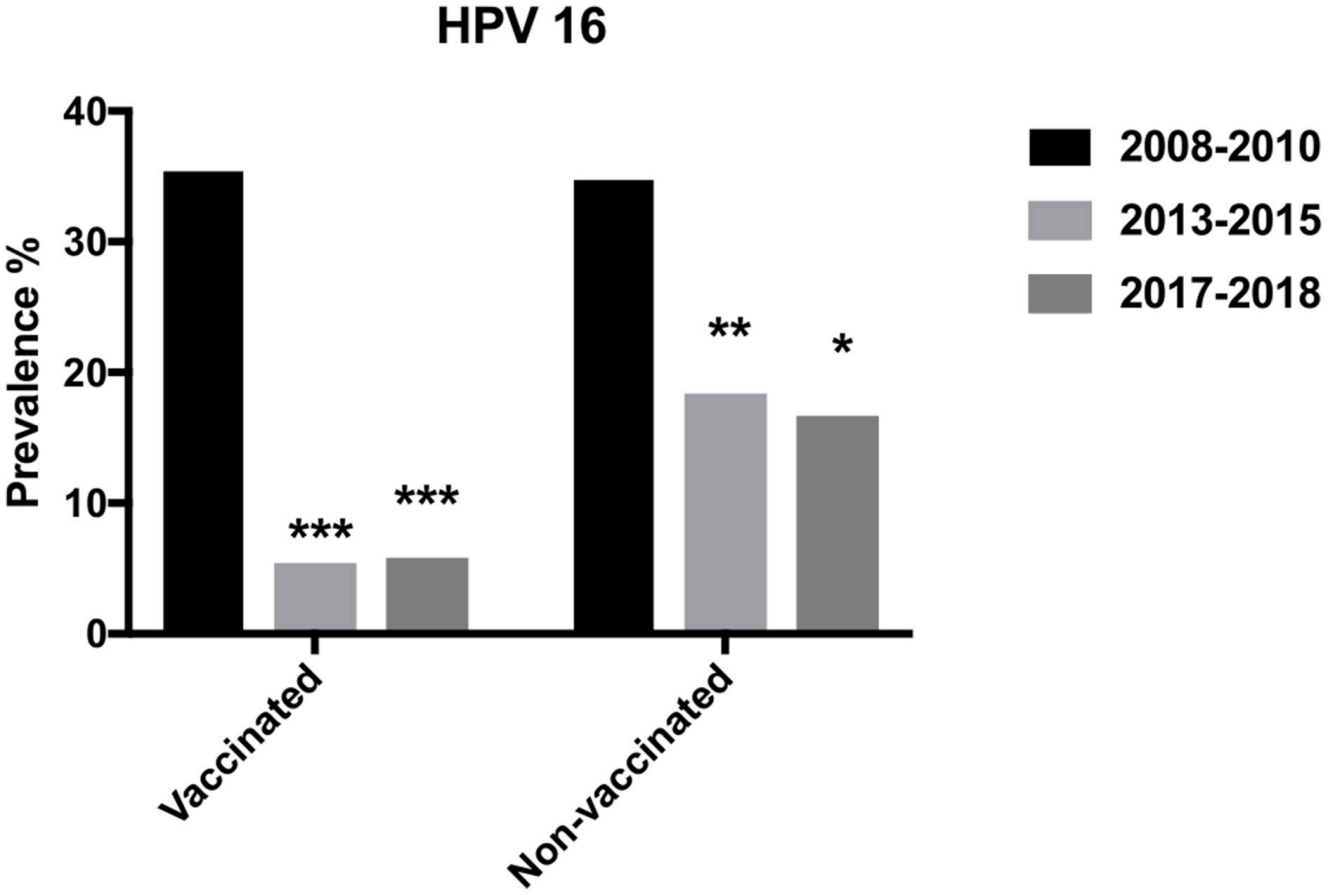
Prevalence of HPV16 in vaccinated and non-vaccinated women over the years 2008–2010, 2013–2015, and 2017–2018. ^*^p < 0.05, ^**^p < 0.01, ^***^p < 0.001.

HPV types included in the vaccination program substantially decreased over time especially in vaccinated women, but also in non-vaccinated women as shown for HPV16 in further detail in Figure 5. During 2017–2018 and 2013–2015 HPV16 prevalence was similar with 5.8 and 5.4%, respectively in vaccinated vs. 16.7 and 18.4%, respectively in non-vaccinated women, while 2008–2010, 35.4% of vaccinated (earlier unpublished data) vs. 34.7% of unvaccinated women were HPV16 positive (Ramqvist et al., 2011; Grün et al., 2016). Prevalence of HPV18, 6, and 11 also tended to decrease over time. HPV18, 6, and 11 were not found in vaccinated women 2017–2018, while in non-vaccinated women, HPV18 and 6 prevalence was 3.3 and 10.0%, respectively, with HPV11 not detected. Notably, 2008–2011, in unvaccinated women prevalence of HPV18, 6, and 11 was 10.1, 8.1, and 2.0%, respectively, while during 2013–2015, corresponding figures were 4.1, 5.1, and 2.0% in non-vaccinated vs. 1.3, 0.4, and 0.4% in vaccinated women (Grün et al., 2016).

### Prevalence of Top Five Non-vaccine HR-HPVs in 2017–2018 Compared to 2008–2010 and Cervical Cancer in 2003–2008

**Figure 6:**
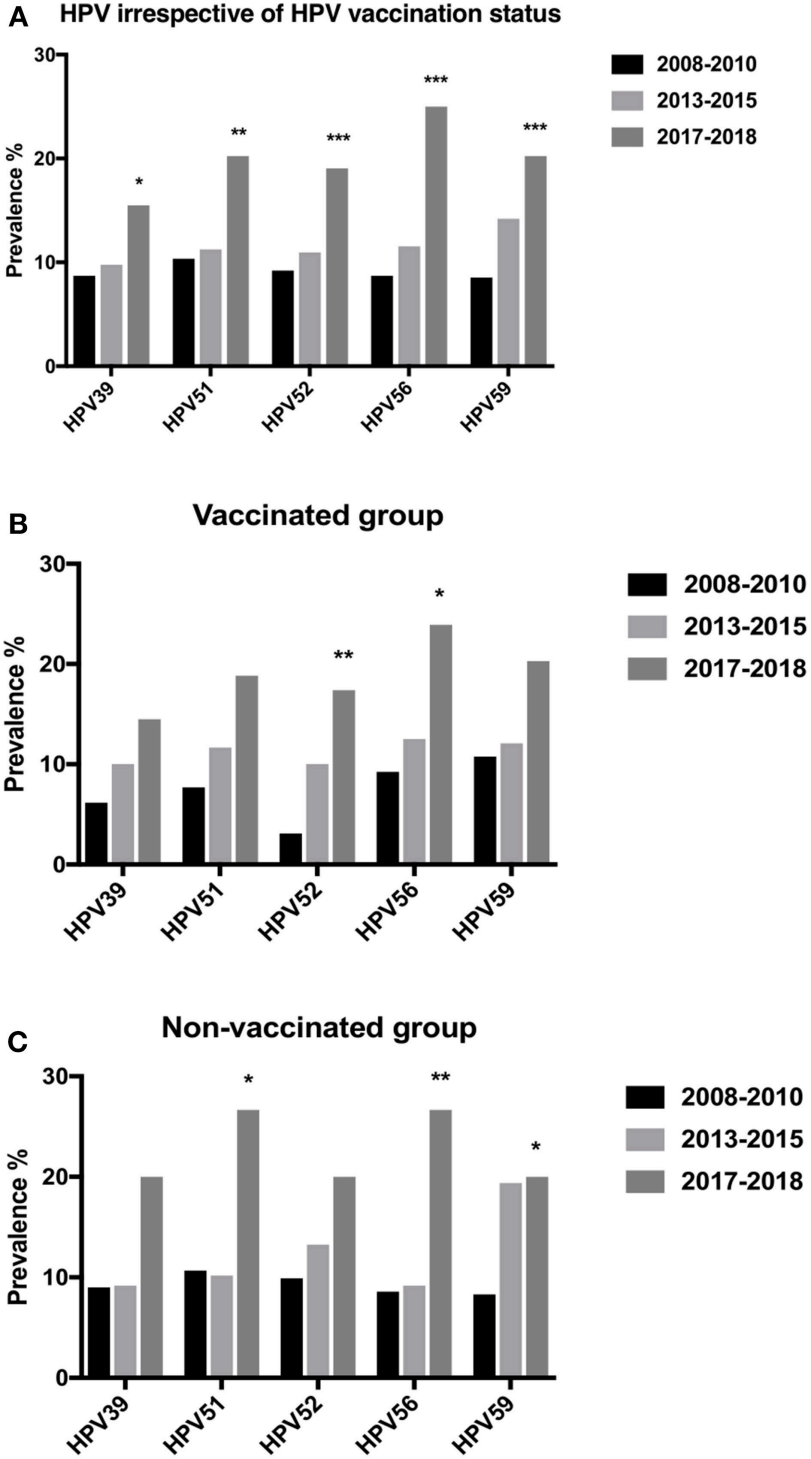
Prevalence of specific human papillomavirus (HPV) types over the years 2008–2018. **(A)** Cervical prevalence of HPV39, 51, 52, 56, and 59 irrespective of vaccination status over the years 2008–2010, 2013–2015, and 2017–2018. **(B)** Cervical prevalence of HPV39, 51, 52, 56, and 59 in vaccinated women over the years 2008–2010, 2013–2015, and 2017–2018. **(C)** Cervical prevalence of HPV39, 51, 52, 56, and 59 in non-vaccinated women over the years 2008–2010, 2013–2015, and 2017–2018. ^*^p < 0.05, ^**^p < 0.01, ^***^p < 0.001.

When comparing (irrespective of vaccination status) HPV-prevalence 2017–2018 of the five most common HR-HPVs (HPV39, 51, 52, 56 and 59) with their corresponding prevalence in 2008–2010, we observed significant increases for all of them (*p* = 0.0138, *p* = 0.0014, *p* = 0.0008, *p* < 0.00001, and *p* = 0.0001, respectively) (Figure 6A). More specifically, in vaccinated women there was a significant increase between 2008–2010 (previously unpublished data) and 2017–2018, for HPV52 and 56 (*p* = 0.0032 and *p* = 0.0132, respectively), while in unvaccinated women, the corresponding rise was significant for HPV51, 56 and 59 (*p* = 0.0147, *p* = 0.0047 and 0.0411, respectively) (Figures 6B,C). Notably, the five most common HR-HPVs, HPV39, 51, 52, 56, and 59 were detected in 14/143 (9.8%) cervical cancer cases 2003–2008, in Stockholm, Sweden (Du et al., 2011).

## Discussion

HPV vaccination, with high coverage has been shown to have an impact on HPV related diseases, reducing the burden of HPV infection, genital warts and cervical disease (Schmitt et al., 2006; Nordfors et al., 2014; Dalianis et al., 2015; Dehlendorff et al., 2018). Here, HPV-prevalence was examined in cervical samples collected 2017–2018 at a Stockholm youth clinic, with previously documented high pre-vaccine HR-HPV-prevalence (62%) (Ramqvist et al., 2011). Acquired data were then compared to HPV-prevalence data obtained at the same clinic the past 10 years (more specifically 2013–2015 and 2008–2010), throughout the introduction of the quadrivalent-Gardasil® vaccine, as well as to HPV-prevalence data in cervical cancer cases 2003–2008, in Stockholm (Du et al., 2011; Ramqvist et al., 2011; Grün et al., 2016). Between 2008 and 2018, the coverage of HPV vaccinated women increased from 10.7%, 2008–2010, to 82.1%, 2017–2018, while the prevalence of HPV16, 18, 6 and 11, the four vaccine types decreased substantially (Ramqvist et al., 2011). During 2017–2018, their combined presence was also significantly lower in vaccinated than in non-vaccinated women. Notably, HPV16 prevalence had also declined in unvaccinated women in 2013–2015 and 2017–2018 as compared to 2008–2010 (Grün et al., 2016). Finally, 2017–2018, HR-HPVs as a whole, and especially HR-HPV35 and 45 decreased in vaccinated as compared unvaccinated women. This did however not apply to the most common HR-HPVs HPV 39, 51, 52, 56, and 59, which instead tended to increase in prevalence 2017–2018 compared to 2008–2010 (Ramqvist et al., 2011). Finally, in the present study, similar to that performed 2008–2010, HPV-prevalence peaked at 21 years of age, and most women with HPV-positive samples had more than one HPV type per specimen (Ramqvist et al., 2011).

These data emphasize the importance of HPV-vaccination and show similar to others studies that high vaccine coverage prevents HPV infection, especially of the four quadrivalent-Gardasil HPV types, and potentially prevents a considerable proportion of HPV related diseases e.g., cervical cancer (Cutts et al., 2007; Carozzi et al., 2018; Dehlendorff et al., 2018; Feiring et al., 2018; Herweijer et al., 2018; Patel et al., 2018; Saccucci et al., 2018; Wei et al., 2018). This is supported by the fact that the combined presence of the four vaccine types was <10% in women below 21 years of age, where vaccine coverage was high (>80%). Furthermore, during 2017–2018, HPV16 was detected in 5.8% of vaccinated women, while HPV 18, 6 and 11 were not detected at all. In addition, due to herd immunity, the prevalence of HPV16 had decreased substantially also in non-vaccinated women when compared to 2008–2010. The latter may along with the limited number of cases partially also explain why the difference in cervical HPV16 prevalence 2017–2018, although lower in vaccinated women than in non-vaccinated women, did not reach statistical significance.

The fact that some HR-HPV types seem to have increased could be due to various reasons. One unconfirmed explanation would be an increased sexual activity in the past decades. Alternatively, one could argue that potential cross-immunization induced by naturally occurring HPV-vaccine types e.g., HPV16, 18, was better against these non-HR HPV types than that induced by the vaccine. This argument was however not supported by the work of Saccucci et al. where e.g., cross-immunization against types genetically related to HPV16 was observed in vaccinated, but not in non-vaccinated women (Saccucci et al., 2018). Elsewise, as suggested by many, the elimination of HPV16, that often produces a strong signal, may have increased the detection rate of non-vaccine HR-HPV types competing for the same primers (Tota et al., 2013; Machalek et al., 2018; Saccucci et al., 2018). Nonetheless, irrespective of the reason, our findings that some HR-HPV types unrelated to those included in the vaccines tend to increase is similar to that reported by others and should be noted and followed, despite the fact that so far it has been argued that it is not due to HPV-type replacement (Ding et al., 2017; Machalek et al., 2018; Saccucci et al., 2018).

Both this study, and our study from the period 2008–2010 (Ramqvist et al., 2011), showed that HPV-prevalence peaked at 21 years of age indicating a relation to sexual activity in line with that observed by others, where HPV-prevalence is high in women younger than 25 years of age (Machalek et al., 2018). Notably, there was higher vaccine coverage in the younger than in the older groups, and this was likely due to the school-based vaccination program introduced in 2012 in Sweden, paralleling data also reported by others (Feiring et al., 2018; Machalek et al., 2018). Moreover, in younger women, the combined prevalence of the HPV-vaccine types was lower (<10%) than in older women, even though the total number of HPV infections dropped after 21 years of age, underscoring the importance of the school-based HPV-vaccination program (Feiring et al., 2018; Machalek et al., 2018).

Earlier, cross-protections induced by the HPV-vaccines were reported against HPV31 and 45, types closely related to HPV16 and 18, respectively (Saccucci et al., 2018; Wei et al., 2018). In this study, HPV35 and 45 showed a much lower prevalence in vaccinated compared with that in non-vaccinated women, suggesting the possibility of cross-immunization also for HPV35 (Saccucci et al., 2018; Wei et al., 2018). Other HPV types with a tendency of lower prevalence in the vaccinated group were HPV 26, 31, 33, 43, 53, 70, and 73, possibly also related to cross-reaction or protection by the vaccine. However, one should not draw hasty conclusions regarding cross-protections, since in other studies with larger cohorts much more heterogeneous outcomes can be observed (Mesher et al., 2016).

Gardasil®9 is the latest HPV-vaccine and covers HPV6, 11, 16, 18, 31, 33, 45, 52, and 58 (Schiller and Lowy, 2018). It includes one (HPV52), but not the other four (HPV39, 51, 56, and 59) most common HR-HPV types found in 2017–2018 at the youth clinic, but it does include five of the six most common HR-HPV types (HPV16, 18, 31, 33, 45, and 56) identified in cervical cancer 2003–2008 in Stockholm (Du et al., 2011). Irrespective of whether HR-HPV types HPV39, 51, 52, 56, and 59, have increased or not from 2008–2010 to 2017–2018, their presence should be cautiously noted, since they together accounted for 9.8% of cervical cancer cases 2003–2008 in Stockholm (Du et al., 2011). Therefore, even if introducing Gardasil®9 one must bear in mind that four of these five HPV types HPV39, 51, 56, and 59 (not included in Gardasil®9), during 2003–2008 contributed to 11/143 (7.7%) of the cervical cancer cases in Stockholm (Du et al., 2011). Notably, although only 6/11 of these were single infections and others were co-infection with another HPV type, even in tumors with co-infections, viral oncogenes of several HPV-types can be transcribed simultaneously (Du et al., 2011; Halec et al., 2013). Furthermore, some potential HR-types not included in Gardasil®9, e.g., HPV53 and HPV66 had a prevalence above 10%, and have as shown for HPV66, been demonstrated as a single infection and actively transcribed in cervical cancer (Halec et al., 2013).

Finally, this study has some bias and limitations that need to be taken into consideration. The young women subjected for this study visit the healthcare center for birth control advice and for the treatment of sexually transmitted diseases (Ramqvist et al., 2011; Grün et al., 2016). Our study, may therefore, be based on a selected group of sexually active young women with possible symptoms for sexually transmitted diseases. Moreover, the potential HR-HPV types HPV 30, 67, 69 assayed in this study, were not tested in our initial study (Ramqvist et al., 2011). Therefore, the total HPV infection rate may have been affected in the investigation over time, while the remaining analyses concerning specific HPV-vaccine or non-vaccine HR-HPV types should not have been influenced by the introduction of these additional types, since these were included previously (Du et al., 2011; Ramqvist et al., 2011).

To conclude, quadrivalent-Gardasil® vaccination has significantly decreased the vaccine specific HPV-types HPV16, 18, 6, and 11, but five non-vaccine specific HR-HPV types HPV39, 51, 52, 56, and 59 still remain high in potentially high-risk women at a youth clinic in Stockholm. Of these five HR-HPV types, only HPV52 is included in Gardasil®9. It is therefore important to follow up HPV infections in the future and possibly consider the most prevalent HPV types in this study for the next generation of HPV-vaccines.

## Data Availability

All datasets generated for this study are included in the manuscript and/or the supplementary files.

## Author Contributions

LE, TD, and JD: conception and design; AÄ-R, TR, LE, TD, and JD: development of methodology; AÄ-R, NG, RU, LH, YH, MS, and AP: acquisition of data; AÄ-R, LC, TR, TD, and JD: analysis and interpretation of data; LC, TD, and JD together with all authors: writing, review, and/or revision of the manuscript.

### Conflict of Interest Statement

The authors declare that the research was conducted in the absence of any commercial or financial relationships that could be construed as a potential conflict of interest.
